# A Rare Cutaneous Adnexal Tumor: Malignant Proliferating Trichilemmal Tumor

**DOI:** 10.1155/2015/742920

**Published:** 2015-05-04

**Authors:** Omer Alici, Musa Kemal Keles, Alper Kurt

**Affiliations:** ^1^Department of Pathology, Medical School, Gaziosmanpasa University, 60300 Tokat, Turkey; ^2^Department of Plastic and Reconstructive Surgery, Medical School, Ondokuz Mayıs University, 55270 Samsun, Turkey; ^3^Department of Plastic and Reconstructive Surgery, Samsun Training and Research Hospital, 55100 Samsun, Turkey

## Abstract

Proliferating trichilemmal tumors (PTTs) are neoplasms derived from the outer root sheath of the hair follicle. These tumors, which commonly affect the scalp of elderly women, rarely demonstrate malignant transformation. Although invasion of the tumors into neighboring tissues and being accompanied with anaplasia and necrosis are accepted as findings of malignancy, histological features may not always be sufficient to identify these tumors. The clinical behavior of the tumor may be incompatible with its histological characteristics. Squamous-cell carcinoma should certainly be considered in differential diagnosis because of its similarity in morphological appearance with PTT. Immunostaining for CD34, P53, and Ki-67 is a useful adjuvant diagnostic method that can be used in differential diagnosis aside from morphological findings. In this study, we aimed to present the case of a 52-year-old female patient with clinicopathological features. We reported a low-grade malignant proliferating trichilemmal tumor in this patient and detected no relapse or metastasis in a 24-month period of follow-up.

## 1. Introduction

Proliferating trichilemmal tumor (PTT) was first described using the term “proliferating epidermoid cyst” by Jones in 1966 [1]. These tumors are rare cutaneous neoplasms derived from the outer root sheath of the hair follicle [2]. Their characteristic histological findings are sudden compact amorphous keratinization of the epithelial cells that cover the cyst wall without a granular layer; this phenomenon is called trichilemmal keratinization [2, 3]. PTTs commonly exhibit benign behavior and rarely present a malignant course [2]. A tumor that invades neighboring tissues accompanied with anaplasia and necrosis is described as a malignant proliferating trichilemmal tumor (MPTT) [2, 3]. MPTTs are invasive and metastatic tumors that demonstrate biologically aggressive behavior [3, 4]. Squamous-cell carcinoma (SCC) should be eliminated by differential diagnosis [2].

In this study, we presented a case with clinicopathological features based on the literature data and reported it as a low-grade MPTT.

## 2. Case Presentation

**Figure 1:**
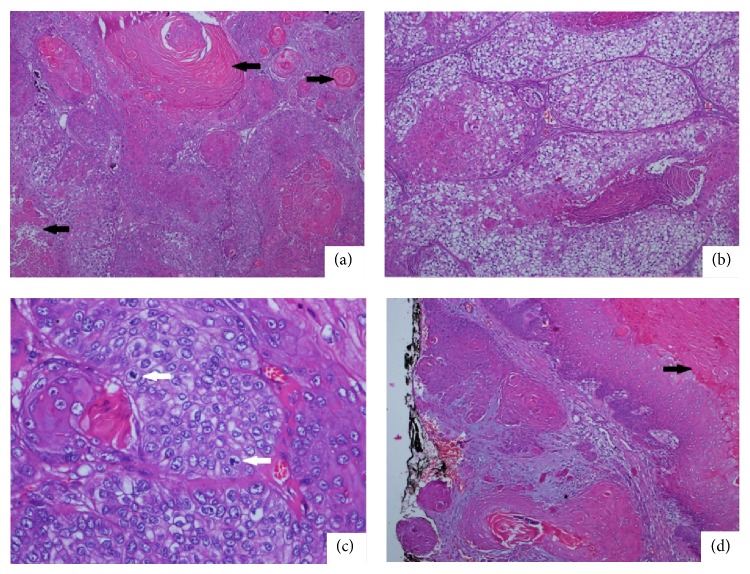
A case of malignant proliferating trichilemmal tumor (H&E, ×100). Tumor cells designed in solid groups are observed. Trichilemmal keratinization (black arrow) at the center of the tumor islands is remarkable (a). The tumor cells with wide translucent cytoplasm are observed more remarkably (b). Atypical mitotic figures in the tumor are remarkable (white arrow) (c). A focal invasion field in the peripheral tumor is observed. The tumor is continuous along the surgical margin labeled by black tissue stain. The focus is on trichilemmal keratinization in the tumor (black arrow) (d).

**Figure 2:**
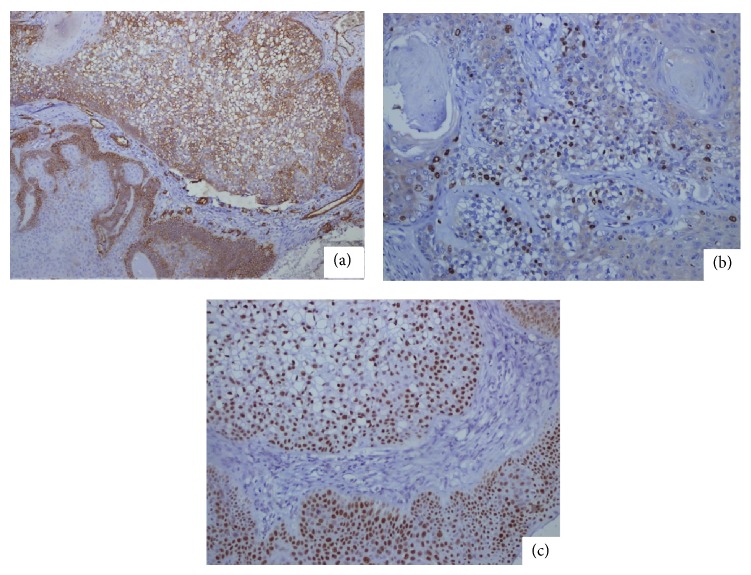
Immunochemical study of the malignant proliferating trichilemmal tumor. Focal CD34 staining is remarkably observed in the cells with translucent cytoplasm and basaloid fields (DAP, ×100) (a). Nuclear Ki-67 staining is remarkably observed in the tumor cells with translucent cytoplasm (DAP, ×200) (b). A diffused and strong staining with P53 is observed (DAP, ×200) (c).

A 52-year-old female patient presented to the Clinic of Plastic Surgery because of three nodular masses on her scalp. The nodular lesions were localized at the occipital, parietal, and sagittal regions with diameters of 5, 3, and 2.5 cm, respectively. No ulceration was observed on the surface of the lesions. The patient reported that the masses had been existing for four years. The dimension of the mass in the occipital region had increased recently, but those of the others did not change. The patient described no trauma or chronic irritation. The patient underwent surgery, and the three excised mass materials were sent to the pathology clinic. The examination of the specimen revealed that the masses excised from the parietal and sagittal regions were macroscopically and microscopically compatible with trichilemmal cyst. The mass excised from the occipital region consisted of a brown-colored nodular lesion with an irregular granular surface with cream-colored fields on top. The section surface of the material was composed of solid and cystic fields that were cream colored. The histopathological evaluation found solid islands standing back-to-back in a desmoplastic basement and a neoplastic formation composed of epithelial cells that were designed in a cordon-like structure and that demonstrated moderate degree of pleomorphism and atypia. The tumor cells commonly had a wide eosinophilic cytoplasm (Figure 1(a)). The rare presence of tumor cell groups with wide translucent cytoplasm was also marked (Figure 1(b)). The tumor included foci of trichilemmal keratinization. Necrosis was not found. Increased atypical mitotic figures, which were more intensely found in the peripheral fields, were present (Figure 1(c)). Mean mitotic count was 10/10 HPF. The tumor was observed to form a relatively regular margin with peripheral fields, partly elongate to the peripheral fields in small infiltrative foci, and exceed the surgical margin (Figure 1(d)). The immunohistochemistry test revealed 40% membranous staining with CD34 in the tumor cells (Figure 2(a)). The Ki-67 proliferation index was approximately 20% (Figure 2(b)). P53 staining was strongly positive in 80% of the cells (Figure 2(c)). According to the morphological findings, the lesion was a low-grade MPTT. The patient underwent a reexcision procedure at the Clinic of Plastic Surgery. No tumor was encountered in the reexcision material within the surgical margin. No relapse or metastasis was detected during a 24-month follow-up of the patient.

## 3. Discussion

PTTs are cutaneous neoplasms generally found as a nodular mass in the scalp of elderly women [3]. These tumors develop in the course of time from the foci of proliferating epithelial cells in the trichilemmal cysts because of factors such as trauma or chronic inflammation [2, 3]. As in our case, the commonly found accompaniment of PTTs with simple trichilemmal cysts may be a supportive finding to be considered [2]. Alternatively, some researchers have suggested that these tumors emerge de novo from organoid nevi [5]. MPTTs are rarely found. Although the presence of high mitotic activity, nuclear polymorphism, tumoral invasion to neighboring tissues, or metastasis can be identified as MPTT, the clinical behavior of the tumor may be incompatible with its histological characteristics in cases with PTT [2, 3]. Cases with good prognosis despite remarkable cytological atypia, as well as patients who died because of metastatic disease although they did not present an infiltrative growth pattern, have been reported in the literature [3, 6]. Accordingly, Ye et al. [4] classified PTTs into three groups, namely, benign, low-grade malignant, and high-grade malignant lesions, based on clinicopathological characteristics. Group I PTTs are benign lesions. Recurrence is not observed in these lesions. They show histologically regular contours with surrounding tissues and mild nuclear atypia but do not involve increased mitotic activity, necrosis, and lymphovascular invasion. Group II PTTs are low-grade malignant tumors. Local recurrence may be observed in these lesions. These tumors have histologically irregular and local invasive contours, and they elongate to the deep dermis and subcutaneous tissue. Group III PTTs are high-grade malignant tumors reported to exhibit a high recurrence rate, lymph nodes, and tendency to develop distant metastasis. Remarkable nuclear pleomorphism, atypical mitosis, necrosis, and lymphovascular invasion may be observed in these tumors. On the basis of this classification, our case can be classified as low-grade MPTT because of the generally moderate degree of atypia and pleomorphism, aside from the absence of lymphovascular invasion and necrosis, despite increased mitotic activity and local infiltrative irregular contours. The absence of a local or distant organ metastasis and recurrence despite a 24-month follow-up clinically supports this conclusion.

SCC should be considered because of its similarity with MPTT in terms of morphological characteristics in differential diagnosis [2, 3]. Trichilemmal keratinization is an important indicator in differentiating PTTs from SCCs [3].

Immunohistochemical studies have also been conducted for the diagnosis and differential diagnosis of PTTs and morphological descriptions [2, 7–9]. Immunohistochemical indicators, such as Ki-67, P53, and CD34, have been given much focus [2, 7–9]. Increased staining with Ki-67 and P53 was observed when MPTT cases were compared with PTT cases in these studies [2, 8]. Ki-67 expression was approximately 20% to 40% in MPTTs, whereas Ki-67 expression was below 5% at the basal layer level in PTTs [2, 8, 9]. The expressions of Ki-67 and P53 at 20% and 80%, respectively, in our case support the literature findings. CD34 is an important immune determinant in the differentiation between SCC and MPTT [2]. It indicates trichilemmal differentiation and is known to be expressed in MPTTs [2, 10]. By contrast, SCCs do not show CD34 expression [2]. Although this difference in staining may be an important characteristic in the elimination of SCCs, negative staining with CD34 was detected in all of the cases in a study that evaluated three distinct MPTT cases [7]. The researchers interpreted this difference in staining with CD34 in MPTT cases to be associated with the differentiation grade of the tumor and the increase in the loss of CD34 staining as the differentiation grade decreases [2].

In conclusion, MPTTs may be confused with SCCs because both have similar morphological characteristics and are rare cases. In the same manner, MPTTs should be accurately distinguished from PTTs. An accurate differential diagnosis is crucial because it will affect the treatment approach for the patient. An immunohistochemical panel composed of CD34, Ki-67, and P53 may be useful in differential diagnosis and determining morphological characteristics. Another issue is that the morphological characteristic of PTTs may not always be compatible with the clinical presentation. Therefore, the patient should be clinically followed up.
